# Antimutagenic Properties of Biologically Active Substances of Microalgae Associates

**DOI:** 10.5195/cajgh.2014.162

**Published:** 2014-12-12

**Authors:** Saule Kolumbayeva, Saule Dzhokebayeva, Dinara Begimbetova, Anna Lovinskaya

**Affiliations:** 1 Department of Biology and Biotechnology, Al-Farabi Kazakh National University, Almaty, Kazakhstan; 2 Ecology Institute, Al-Farabi Kazakh National University, Almaty, Kazakhstan; 3 Center for Life Sciences, Nazarbayev University, Astana, Kazakhstan

**Keywords:** natural protectors, microalgae, biologically active polypeptide, antimutagenic activity

## Abstract

**Introduction:**

There are an increasing number of different xenobiotics negatively influencing population health. Therefore, it is important to find effective protectors against mutagenic and toxic effects of environmental pollutants. Naturally occurring biologically active substances, the majority of which are antioxidants, are capable of functioning as modifiers of the induced mutation process. The application of various naturally occurring protectors will lower essential risks of congenital malformations, cancer, and hereditary diseases caused by mutational damages. Therefore, it is crucial to screen algal flora of Kazakhstan for the antimutagenic activity. This study involved the assessment of antimutagenic potential of biologically active polypeptide (BAP) produced in mixed microalgae cultures.

**Methods:**

70 white outbred male rats (Rattus norvegicus) at 6 months of age were used for this study. The dosage of BAP produced by microalgae associates Anabaena flos-aquae x Anabaenopsis sp. comprised 100 mg/kg. Cadmium sulfate was used as a mutagen in a concentration of 1 mg/kg. Experiments on antimutagenic activity of BAP were carried out with the Mammalian Bone Marrow Chromosomal Aberration Test.

**Results:**

After acute and subacute exposure of BAP, the level of chromosomal structural abnormalities in rat bone marrow cells was the same as in control group. Therefore, BAP showed no mutagenic activity, whereas exposure to cadmium sulfate at used concentration induced chromosomal aberrations with a significantly higher frequency than the spontaneous mutation rate. The exposure combination of BAP with cadmium sulfate resulted in a two-fold decrease (*p*< 0.05) of mutagen-induced chromosomal aberrations. The range of induced chromosomal aberrations included alterations of all types both in control and experimental groups.

**Conclusion:**

Most of the genotoxic effects are mediated through oxidative stress. The repair of DNA damage is an enzymatic process, which depends on the cellular metabolic rate. It has previously been shown that many biologically active substances lead to reduction of DNA sensitivity to mutagenic damaging factors. Based on these facts and obtained results, it can be assumed that BAP from mixed microalgae cultures Anabaena flos-aquae x Anabaenopsis sp. are capable of blocking free radical process reducing the likelihood of genome damage, as well as triggering the cellular repair system.

